# Can Prostate Cancer Screening Reduce Mortality in Men Aged 75–79 Years? A Protocol for Target-trial Emulation

**DOI:** 10.1016/j.euros.2025.10.016

**Published:** 2025-11-06

**Authors:** Ahmad Abbadi, Chiara Micoli, Andrea Discacciati, Camila Olarte Parra, Anna Lantz, Lars Björnebo, Markus Arvendell, Jan Chandra Engel, Joel Andersson, Ugo Falagario, Henrik Grönberg, Martin Eklund, Mark Clements, Tobias Nordström

**Affiliations:** a Department of Medical Epidemiology and Biostatistics, Karolinska Institutet, Solna, Sweden; b Unit of Epidemiology, Institute of Environmental Medicine, Karolinska Institutet, Solna, Stockholm, Sweden; c Department of Molecular Medicine and Surgery (Solna), Karolinska Institutet, Stockholm, Sweden; d Department of Internal Medicine, the University of Pittsburgh Medical Center, Pittsburgh, Pennsylvania, USA; e Department of Urology, Södersjukhuset, Stockholm, Sweden; f Department of Clinical Sciences at Danderyds Hospital, Karolinska Institutet, Solna, Sweden; g Department of Urology and Kidney Transplantation, University of Foggia, Foggia, Italy; h Department of Oncology, Capio S:t Görans Hospital, Stockholm, Sweden

**Keywords:** Prostate cancer, Screening, Prostate-specific antigen, Older adults, Urology, Mortality, Emulation of target trial

## Abstract

Opportunistic testing using the prostate-specific antigen (PSA) test is common among men aged 75–79 yr. American and European guidelines consider PSA-based screening through joint decision-making if men have at least 10 yr of life expectancy. The age threshold for PSA-based screening was previously described in these guidelines as 70–74 yr. However, Swedish men, similar to their global counterparts, are experiencing increased life expectancy. As of 2024, Swedish men aged 75–79 yr have on average >10 yr of life expectancy. To date, there are no randomized controlled trials on prostate cancer screening for men aged 75–79 yr at baseline. With a lack of randomized data, we plan on leveraging the population-based Stockholm Prostate Cancer Diagnostics Register from 2007 to 2023, to explore the effect of PSA-based screening on all-cause and prostate cancer–specific mortality. In this target-trial emulation study protocol, our aim is to evaluate whether PSA-based screening in men aged 75–79 yr with no previous prostate cancer diagnosis would have survival benefits from PSA-based screening. We will conduct sequential and clone-censor-weight target-trial emulations based on the age at enrollment, assigning men to screened or unscreened arms based on the observational analog of an intention-to-screen analysis of PSA test records. Men will be followed until death or December 31, 2023. The statistical analysis involves survival models, reporting of the cumulative incidence functions (CIF), and differences in CIFs. Sensitivity analyses aim to evaluate whether similar findings can be observed if we restrict enrollment to those who have a minimum of 5 and 10 yr of follow-up, consider calendar year instead of age at enrollment, and consider enrollment of younger men. This study aims to provide evidence on the survival benefits of PSA-based screening in this age group, and clarify whether extending screening guidelines to men aged 75–79 yr would reduce mortality without exacerbating the risk of overdiagnosis. Findings will guide clinical recommendations and inform the design of future randomized controlled trials. The study is registered at ClinicalTrials.gov (NCT07206693).

## Take Home Message

This protocol plans to evaluate whether prostate-specific antigen (PSA)-based screening reduces mortality in men aged 75–79 yr using target-trial emulation, to inform future screening guidelines and randomized controlled trials.

## 1 Introduction

Prostate cancer (PCa) screening allows for the early detection of potentially aggressive PCa cases to reduce mortality and morbidity [1]. Among Swedish men, PCa is the leading cause of cancer mortality, accounting for nearly fifth of all cancer deaths [2]. Currently, prostate-specific antigen (PSA)-based screening is available for men with life expectancy of ≥10 yr, provided that they understand the risks and benefits of PCa screening through a shared decision-making process [3]. Current European Association of Urology and American Urology Association guidelines suggest PSA-based screening up to ages 70 or 74 yr [3,4]. Despite these recommendations, PSA-based screening in men aged ≥75 yr is common [[5], [6], [7]]. As populations live longer [8], the number of older men with at least ≥10 yr of life expectancy is increasing. In 2024, Swedish men aged 75 yr had average life expectancy of 12.44 yr, while those aged 79 yr had average life expectancy of 9.74 yr [9]. With this trend, life expectancy might exceed 10 yr in men older than 79 yr in the near future. Therefore, fit men aged ≥75 yr could be considered for screening according to medical guidelines.

On the contrary, with increasing age, the number and complexity of chronic diseases increase [10]. Septuagenarians have heterogeneous health status declining with increased age [11]. Detection of PCa that is unlikely to metastasize or cause health issues might lead to unnecessary stress or worry, with health outcomes exacerbated negatively by the potential overtreatment [12]. Furthermore, if a PCa case is to be detected, the choice of radical or conservative therapy can have an impact on survival [[13], [14], [15]].

To address the dilemma of screening for PCa among older men, and to provide recommendations on who to test and when to stop screening, randomized controlled trials (RCTs) would provide the strongest evidence. However, even if an RCT were to be conducted specifically to address this gap, the long follow-up necessary for such an RCT to mature would delay the availability of evidence to make clinical and epidemiological recommendations. Moreover, there might be ethical concerns about conducting an RCT without demonstrated benefit leveraging observational data in alignment with the principle of equipoise [16]. The usage of large observational data to emulate a target trial can assist in bridging the gap and provide directions to inform future RCTs [17]. Emulation of target trials aims to address causal questions utilizing several statistical and epidemiological methods to capitalize on available observational data to reach potentially similar findings as RCTs conditional on the proper data structuring and data analysis in line of addressing the research question [[18], [19], [20], [21]].

Hence, in this protocol, we outline the study aims to address two research questions: (1) What are the characteristics of men aged 75–79 yr who are screened despite no clinical recommendations? (2) Utilizing a target-trial emulation, what is the impact of PSA-based screening on all-cause and PCa-specific mortality among men aged 75–79 yr?

## 2 Methods

To address the aims of the study, an intention-to-screen analysis plan of a target-trial emulation will be conducted based on PSA testing among men aged 75–79 yr. The target-trial emulation protocol and study conduct will follow the guidelines and recommendations of Hernán et al [17,20].

### 2.1 Hypothesis

Our hypothesis is that men aged 75–79 yr who are screened for PCa using PSA, despite no prior PCa diagnosis, gain a modest survival benefit by reducing PCa mortality through the detection and future treatment of lesions with metastatic potential.

### 2.2 Study population and data source

**Fig. 1:**
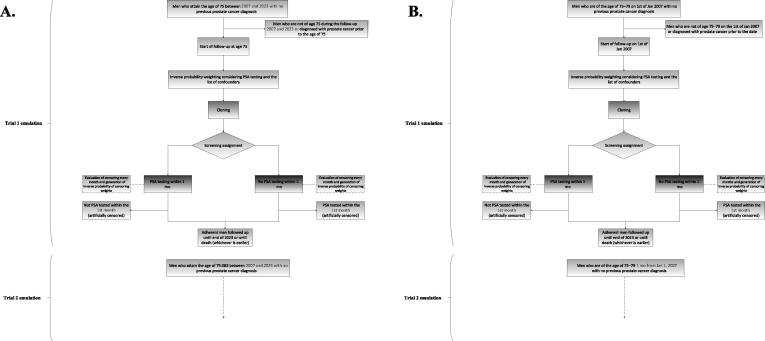
Flowchart showing inclusion into and exclusion from emulated target trials. (A) The main analysis inclusion based on age. (B) The sensitivity analysis based on calendar year. Cloning refers to the duplication of each participant in the study, which is later assigned to one of the two screening strategies. PSA = prostate-specific antigen.

This study and the target-trial emulation will use population-based register data of men living in Stockholm between 2007 and 2023, who were screened for PCa, extracted from the Stockholm Prostate Cancer Diagnostics Register (STHLM0), a comprehensive register of all men tested for PCa with PSA in Stockholm, Sweden [22]. The STHLM0 has complete PSA test records since January 1, 2007 [22]. This will be supplemented with information on nontested men from the STHLM0+ register [22]. Demographic information, family history of PCa, diagnoses, hospitalization, specialized outpatient health care, PCa treatment, and mortality information are provided from the linked Swedish population registers [22]. Figure 1 illustrates the flowchart of inclusion into the target-trial emulation.

### 2.3 Eligibility criteria

Men aged 75–79 yr between 2007 and 2023, with no PCa diagnosis at the time of inclusion (based on each trial emulation), will be considered eligible for the trials. To ensure proper control for potential confounding and to be able to evaluate the eligibility criteria, only men with complete information on their screening status, outcomes, and confounders in the registers will be included. The summary number of the men excluded due to missing values will be described in the final study flowchart.

### 2.4 Screening strategy

Two types of target-trial emulations are proposed, with both aimed to address the second research question but using different causal inference methods. Two different methods are performed to evaluate whether the results would agree, giving confidence to the conclusions made based on the findings of the study. The first method employs a sequential design and the clone-censor-weight (CCW) approach with enrollment/inclusion based on the attained age (main analysis). The second method performs the analysis based on the calendar year (sensitivity analysis). The CCW approach handles time-varying eligibility and treatment assignment while avoiding the immortal time bias, and the sequential design enables repeated trial emulation at multiple entry points to maximize generalizability and emulate real-world decision-making [20,21].

### 2.5 Main analysis—sequential design with the CSW approach based on age at enrollment

Emulated target trials assign men into one of the two screening arms based on their screening pattern during the eligibility period, which starts when they attain the age of interest for the study (75–79 yr) for the first time and every month thereafter (eg, at age 75 yr, 75.083 yr, 75.167 yr, and so on).

Screening is ascertained within the 1st month from trial initiation, representing the eligibility and assignment period. Eligible men with at least one PSA test in this period will be assigned to the screening arm. Those with no PSA test in this window will be assigned to the control arm. Assignment ascertainment is assumed to be accurate for all participants, as the STHLM0 contains the data on PSA testing for men living in Stockholm and who were tested in Stockholm since 2007 [22]. Considering the assumptions of causal inference frameworks [19], the following three principles are assumed to be fulfilled:

1. Positivity: Every man living in Stockholm within the age group of interest has a nonzero chance of deviating from the protocol at any point during the assessment period, and every eligible man is assumed to have had the opportunity to undergo a PSA test.
2. Consistency: The survival outcome we observe for the men under the given screening strategy is the same as the outcome that would have occurred if we had explicitly assigned these patients to follow the same strategy (ie, their potential outcome). As such, the screening protocol is assumed to have been defined clearly and applied consistently throughout the study period in our study. Moreover, we assume that the statistical models used in the analysis (described below) are specified correctly.
3. Exchangeability: There are no unmeasured confounders; all factors that influence both the screening assignment and the likelihood of deviating from the protocol have been measured. We assume based on the confounders accounted for in the study (described below) that we address the potential factors that impact the screening assignment.

### 2.6 Assignment to screening strategies

Since randomization is not possible in the emulation of target trials, men will be assigned to the screening strategy in each of the trials based on their observational data. Considering that the intention of the PSA test is not recorded in the register, additional measures will be taken to ensure proper capture of the PSA-based screening instead of diagnostic PSA testing. Men who have had an immediate diagnosis following their PSA test (ascertained as within 6 mo from the test), and the diagnosis showed distant metastasis (M1) or the diagnosis was prompted by urinary or other symptoms, the PSA test will not be classified as a PSA-based screening test. If the PSA test was preceded by urological symptoms or bone pain within 6 mo in the patient register, the test will not be classified as a PSA-based screening test. Moreover, we will not consider men as screened if they underwent a PSA test, but have no record of PCa diagnosis in the registers, yet died from PCa within 6 mo of the PSA test. We will assume that the adjustment for the confounders is sufficient to mimic the randomization in the target trial (ie, we assume that they are randomly assigned within the levels of the confounders). Considering the observational nature of the study, blinding is not possible, and the men will be aware of their screening activity.

Figure 1A illustrates the flowchart of the inclusion into the trial based on the main analysis design.

### 2.7 Follow-up period

Men will initiate their follow-up based on the time of inclusion, starting from January 1, 2007, and end their follow-up by December 31, 2023, or if they experience the outcome of interest, whichever occurs first. The last recruitment age is 79.91 yr. The date of inclusion (time zero) will depend on the trial design; in the main analysis, this will be the date of age attainment.

### 2.8 Outcomes

The first outcome of interest is all-cause mortality, which will be based on the reported date of death. The second outcome of interest is PCa-specific mortality, which will be ascertained based on the leading cause of death. The International Classification of Disease tenth revision is used to report the cause of death, and the code C61 is used to identify PCa-specific mortality. Men who are alive by December 31, 2023 will be censored by reaching the end of the follow-up period.

### 2.9 Causal contrasts of interest

This study will consider an observational analog of an intention-to-screen analysis, comparing the screening strategies assigned during the ascertainment period. The no screening (control) arm will be used as the reference group in all analyses.

### 2.10 Statistical analysis plan

#### 2.10.1 Data preparation and choice of confounders

Data on all men living in Stockholm will be used, and ascertainment of time zero will be performed for all eligible men with no information on the variables necessary for the analysis missing. As men can continuously be eligible to be included in the sequential trials, the choice of time zero will follow the recommendation of Hernán et al [17], and emulation will be done repetitively during the eligibility period between 2007 and 2023. The confounders of interest are age (continuous), trial year (categorical; from 2007 to 2023), country/region of birth (categorical; Sweden, Nordic, European Union, and others), income (categorical; five quintiles), previous hospitalization during the past year (yes/no), number of hospitalizations during the past year (continuous), number of outpatient specialized health care visits (continuous), non–age-adjusted Charlson Comorbidity Index (continuous, based on adaptations for register-based Swedish data [20], and cumulatively computed, based on the past 5 yr until the end of the previous calendar year before the assessment), education (categorical; ≤9 yr of formal education, 10–12 yr of formal education, and ≥12 yr of formal education), civil status (categorical; not married, married, divorced, and widowed), current cancer diagnosis excluding PCa (yes/no), and family history of PCa (yes/no, based on father, brother[s], or son[s] diagnosed with PCa).

#### 2.10.2 Statistical considerations

Regarding the CCW trial emulation, two arms will be constructed representing the two screening strategies compared in each pair (screening vs no screening). As the period of eligibility is long (age 75–79 yr), it would artificially inflate the benefit of the no screening strategy as it would contribute to more deaths before the time of censoring in the screening arm. Hence, the trials will be conducted every month during the eligibility period (ie, 75–79 yr of age). Those screened in the control arm will be censored at the first date of their screening during the eligibility 1-mo period, while those not screened in the intervention arm will be censored at the end of the eligibility 1-mo period. Since the datasets will be censored artificially, the inverse probability of censoring weighting (IPCW) will be constructed by applying Cox regression using the list of confounders outlined earlier. Considering that the dataset in the STHLM0 is large (>200 000 men in the age group since 2007) [22], it is computationally prohibitive to construct the censoring weights at each censoring event. We will therefore construct these at the end of the eligibility period. All the datasets will then be appended to perform the analysis and will account for the variance from the same individuals contributing multiple times to the analysis.

Any deviation from the analysis plan or the proposed study design will be described and justified in the study publication.

#### 2.10.3 Descriptive statistics

To address the first research question on the characteristics of men who screen for PCa using PSA despite no clinical recommendations, the baseline clinical and demographic characteristics of men who are screened in this age group will be quantified in a descriptive table. Moreover, the outcomes of the screening if resulted in the diagnosis of PCa would be quantified in terms of Gleason group; tumor, node, and metastasis stage; status if preceded by symptoms; and clinical significance (defined for pragmatic reasons as Gleason group ≥3 + 4). Categorical variables will be reported in terms of numbers and percentages, while continuous variables will be reported in term of means and standard deviations, or medians and interquartile ranges if the standard deviation is large.

#### 2.10.4 Survival analysis

The analysis will be conducted using Stata 18 (Stata Corporation, College Station, TX, USA). Flexible parametric survival models will be used to model the log cumulative hazard with natural splines. Selection of the number of degrees of freedom for the baseline hazard will be done by evaluating the Akaike's Information Criteria [23]. Time-dependent coefficients will be used to account for a potential time-varying effect of the screening. Since men can participate in several trials, the variance-covariance matrix will be estimated using the clustered sandwich estimator. The stabilized inverse probability of screening weighting (IPW) will be generated using logistic regression. In presence of extreme weights, IPW will be truncated at the 99th percentile [21] and then multiplied by the IPCW. The product will be used to weigh the likelihood of the flexible parametric models. The user-written command stpm3 will be used to fit the flexible parametric survival models [24].

Standardized model-based cumulative incidence functions (CIFs) will be plotted and reported for all outcomes, and the difference in CIFs will also be plotted and reported at 5, 10, and 15 yr of follow-up. Standardization will be done over the empirical distribution of the confounders using the command standsurv [25]. The CIF difference for both the PCa-specific mortality and the all-cause mortality will be reported.

#### 2.10.5 Subgroup analysis

The analysis will also be stratified by age, and each year group (eg, 75 yr, 76 yr, etc.) will be presented separately. A comparison between the pooled trials and the age stratifications will be described, and whether a potential benefit/no benefit is observed in certain age groups will be reported. Additionally, a subgroup analysis would be performed among men with Charlson Comorbidity Index ≤10, as the group with Charlson Comorbidity Index >10 might rarely be offered PSA-based screening.

### 2.11 Sensitivity analysis

#### 2.11.1 Analysis based on calendar year

Emulated target trials with observational analog of an intention-to-screen analysis will be conducted, using the CCW method and sequential design every month in alignment to the main analysis. However, the month’s assessment will start from January 1, 2007. Men are ascertained for their screening within the month if they are eligible (75–79 yr). The intervention arm includes eligible men screened with PSA for PCa, while the control arm includes eligible men not screened for PCa during that specific month period. Follow-up starts on January 1, 2007 and ends on December 31, 2023, or when mortality occurs, whichever comes first.

Figure 1B illustrates the flowchart of the inclusion into the trial based on the sensitivity analysis.

#### 2.11.2 Other sensitivity analysis considerations

In addition to the different causal inference methods, this study will consider limiting the recruitment period to December 2018 and December 2013 to allow for longer follow-up time among eligible men. We will consider extending the ascertainment period of the PSA-based screening to 12 mo instead of 6 mo. A deterministic sensitivity analysis for unmeasured confounding using the array method, as described by Schneeweiss [26], will be performed, and the curved surface representing the “fully” adjusted ratio based on the assumed prevalence and varied strength of the unmeasured confounder will be plotted. Moreover, the results will be contrasted with men aged 65–69 and 58–62 yr (in contrast to men aged 75–79 yr).

### 2.12 Power calculation

The STHLM0 and STHLM0+ combined have >200 000 men in the eligible age group between 2007 and 2023 [22]. Considering life expectancy of 11.5 yr on average for men aged 75–79 yr and 10 yr for men aged 80 yr, we can assume that, with a minimum of 200 000 men eligible for the study and 12 500 men enrolled each year, around 35 000 deaths would be recorded during the follow-up from 2007 to 2023. Hence, assuming a baseline cumulative mortality of 0.80 after 17 yr and a proportion of screening of 20%, Table 1 summarizes the power according to five different scenarios of potential reduction in mortality. Based on the power calculations, the study is powered sufficiently (>90%) to detect a benefit of at least 1.5% absolute mortality reduction. A minimum of 1.28% absolute risk reduction is necessary to guarantee at least 80% power.

**Table 1:** Power calculations for all-cause mortality

ARR (%)	HR^a^	Power with 35 000 deaths (%)	Minimum deaths needed for 80% power
1.0	0.970	62.5	∼52 700
1.5	0.956	92.0	∼24 200
2.0	0.941	99.6	∼12 800
2.5	0.927	>99.9	∼8500
3.0	0.913	>99.9	∼5930

ARR = absolute risk reduction; HR = hazard ratio.

a Hazard ratios are approximates based on the baseline mortality and the absolute reductions.

### 2.13 Reporting

This study will adhere to the TrAnsparent ReportinG of observational studies Emulating a Target trial (TARGET) reporting guideline [27].

## 3 Discussion

In this study and target-trial emulation, we aim to address crucial questions that remain unanswered by the current RCTs, namely, when should PCa-based screening be discontinued in the context of increasing life expectancy, and is there any benefit in screening men aged 75–79 yr who have no prior PCa diagnosis?

### 3.1 Potential findings and clinical implications

The findings of this study could demonstrate the benefit of PSA-based screening among men aged 75–79 yr, no observed benefit, or increased harm. In the scenario that the study shows a modest benefit, the medical guidelines can be informed about the emerging evidence to contrast it with their current recommendations. The screening age could then be increased to include men aged 75–79 yr on the conditions that the country has high life expectancy within this age group and the patient is not frail. Moreover, the study would provide foundational support to the conduct of a screening RCT aimed at this age group. It also poses questions on how men should be assessed for their health status before providing screening diagnostics [28,29], how to ensure that the PCa screening minimizes overdiagnosis [30], how to reduce overtreatment, and which treatment modality would optimize their survival and recovery after treatment [31]. On the contrary, if the study fails to show a benefit despite the long-term follow-up, or shows increased harm, the medical guidelines should then consider strengthening the recommendation about no screening after the age of 74 yr, and the importance of urologists’ re-engagement and communication with the patients in the shared decision-making session.

### 3.2 Reflection on RCTs and other observational studies

Prior RCTs attempted to quantify the benefit of PCa screening in terms of PCa-specific and all-cause mortality, but these trials focused on younger populations with distinct study designs and objectives [[32], [33], [34]]. Our study design reflected upon the RCTs performed previously, namely, the Cluster randomised triAl of PSA testing for Prostate cancer (CAP) [33] and the European Randomized Study of Screening for Prostate Cancer (ERSPC) [34]. However, in their design, the age of interest was different, as the CAP randomized trial included men aged 50–69 yr and invited them for a single PSA test [35], while the ERSPC included primarily men of age 55–69 yr [36], with some centers extending the range to 50–54 and 70–74 yr [34]. In the CAP study, an intention-to-screen analysis was performed and a modest benefit was found for PCa-specific mortality in the screened group with 15 yr of follow-up [35]. The ERSPC study group conducted both intention-to-screen analyses and analyses to account for the contamination and noncompliance to their study, which after adjustment showed higher reduction of PCa mortality following screening [36]. With long-term follow-up and having men in the control arms of RCTs performing opportunistic testing during the time of follow-up dilute the potential benefits of the intervention and make the two arms more similar [37]. In the two designs proposed, we allow men to vary in their status based on each trial conducted, and then account for the intercluster correlation.

The observational study by García-Albéniz et al [38] described, in their target-trial emulation, a benefit of annual PSA-based screening compared with no screening among men aged 67–74 yr, but it was not statistically significant among men aged 75–84 yr. However, the follow-up period was limited to 8 yr in their study [38]. Longer follow-up might provide an opportunity for the emulated trials to show a better survival benefit among the screened men [38].

### 3.3 Potential strengths and limitations

Our study will leverage the STHLM0, which has near complete information on all PSA tests in Stockholm, with linkage to national Swedish registers allowing the capture of diverse lists of potential confounders [22]. In our planned study, we consider the emulation of pragmatic target trials suitable for addressing the research questions at hand. Moreover, the potential follow-up of up to 17 yr can provide sufficient time for the relevant outcomes to occur. Henceforth, this work has the potential to guide decisions on PCa screening cessation, addressing an important need among men who undergo opportunistic testing despite the lack of guidelines for this age group.

Despite these considerations, the study has potential limitations. First, our analysis plan assumes adherence to the assigned arm at each trial, which might not be ideal. Some of the men who will be considered as screened might have been prompted to undergo a PSA test following the symptoms of metastasis or urinary retention. However, the cause of the PSA test cannot be discerned from the register data. The diagnosis if preceded by symptoms is captured in the register but has incomplete information [39]. Additionally, men who underwent a PSA test within the study period due to health checkup following previously elevated tests are classified as screened in the study. However, due to the shift to organized PCa testing [40], this approach might better reflect future screening directions of continuing screening in this age group that was initiated at an earlier age, instead of initiating screening in this age group. Second, unmeasured and residual confounding cannot be ruled out, and data on functionality, holistic objective health status, and disability are not available from the registers. Although we attempt to conduct a subgroup analysis based on the level of multimorbidity excluding those who are sickest, it would not granularly capture individual causes of the PSA testing. Additionally, men who are institutionalized might not have admission or usage of specialized outpatient care, underestimating their multimorbidity.

The actual capture of the outcome in PCa-specific mortality might be challenging to ascertain without autopsy among older men as the quality of the cause-of-death reporting decreases with age, and the death certificate might be suboptimal to capture PCa mortality among the oldest age group [[41], [42], [43]]. This observation has been reported in Nordic countries [42,44]. Thus, the sticky-diagnosis bias might affect determining whether PCa was the cause of death among the screened men compared with the nonscreened men [45]. As such, evaluation of both all-cause and PCa-specific mortality would be warranted, and this misclassification could affect PCa mortality, but not all-cause mortality [46]. A comparison of the risks among younger age groups can assist in ascertaining whether a potential difference in direction is due to the limitations of death certificates in older age and the sticky-diagnosis bias.

Although the study focuses primarily on mortality outcomes, diagnosis outcomes are addressed only descriptively, and the quality-of-life outcomes are not available in the registries. These findings would be needed for a fully informed decision-making process and subsequent updates to clinical guidelines.

Positivity is assumed as part of the emulation of target-trial design [47]. However, this might not be feasible in real life, as men who are very frail would be evaluated differently by health care providers and might not be considered for screening by the urologist as they might not be able to tolerate even conservative therapy.

## 4 Conclusions

With increasing life expectancy, older men face complex decisions about PCa screening, raising questions about whether and when they should continue PSA-based screening to balance the potential benefits in reducing PCa mortality against the risks of overdiagnosis. In this target-trial emulation study protocol, we outlined the study rationale, hypothesis, and research questions, along with the design, analysis plan, and a careful assessment of potential strengths and limitations. This project seeks to provide meaningful insights into these concerns, offering guidance to physicians and supporting informed, shared decision-making with their patients.

  ***Author contributions:*** Ahmad Abbadi had full access to all the data in the study and takes responsibility for the integrity of the data and the accuracy of the data analysis.

  *Study concept and design*: Abbadi, Nordström, Micoli, Discacciati, Olarte Parra, Eklund, Clements.

*Acquisition of data*: None.

*Analysis and interpretation of data*: None.

*Drafting of the manuscript*: Abbadi, Nordström.

*Critical revision of the manuscript for important intellectual content*: All authors.

*Statistical analysis*: None.

*Obtaining funding*: Abbadi, Nordström.

*Administrative, technical, or material support*: None.

*Supervision*: Nordström.

*Other*: None.

  ***Financial disclosures:*** Ahmad Abbadi certifies that all conflicts of interest, including specific financial interests and relationships and affiliations relevant to the subject matter or materials discussed in the manuscript (eg, employment/affiliation, grants or funding, consultancies, honoraria, stock ownership or options, expert testimony, royalties, or patents filed, received, or pending), are the following: Tobias Nordström owns stock in A3P Biomedical AB. Henrik Grönberg holds patents and owns stock in A3P Biomedical AB. Martin Eklund holds patents in A3P Biomedical AB, and reports being a stockholder of A3P Biomedical AB and Clinsight AB. The remaining authors have nothing to disclose.

  ***Funding/Support and role of the sponsor:*** Ahmad Abbadi has received funding from Stiftelsen Sigurd och Elsa Goljes Minne (LA2025-0004). Tobias Nordström received funding from the Swedish Cancer Society (Cancerfonden), the Swedish Research Council (Vetenskapsrådet), the Swedish Research Council for Health Working Life and Welfare (FORTE), the Strategic Research Programme on Cancer (StratCan), Hagstrandska Minnesfonden, Region Stockholm, Svenska Druidorden, Åke Wibergs Stiftelse, the Swedish e-Science Research Centre, the Karolinska Institutet, and Prostatacancerförbundet. The funders had no role in the design and conduct of the study; collection, management, analysis, and interpretation of the data; preparation, review, or approval of the manuscript; and decision to submit the manuscript for publication.

  ***Data sharing statement:*** The datasets generated and/or analyzed in the current study are not publicly available due to ethical and data sharing restrictions/laws, including but not limited to GDPR. The data, however, can be requested formally from the principal investigator of each dataset.

  ***Ethics statement:*** STHLM0 was approved by local Ethics Committee at Karolinska Institutet and regional Ethics Board in Stockholm (Dnr: 2012/438-31/3), and the planned study is covered by that ethics committee approval and its amendments. All the research conducted and planned conforms to the Declaration of Helsinki. The study is registered at ClinicalTrials.gov (NCT07206693).
